# Predicting treatment response to neoadjuvant chemoradiotherapy in local advanced rectal cancer by biopsy digital pathology image features

**DOI:** 10.1002/ctm2.110

**Published:** 2020-06-28

**Authors:** Fang Zhang, Su Yao, Zhi Li, Changhong Liang, Ke Zhao, Yanqi Huang, Ying Gao, Jinrong Qu, Zhenhui Li, Zaiyi Liu

**Affiliations:** ^1^ School of Computer Science and Engineering South China University of Technology Guangzhou Guangdong China; ^2^ Department of Radiology Guangdong Provincial People's Hospital, Guangdong Academy of Medical Sciences Guangzhou Guangdong China; ^3^ Department of Pathology Guangdong Provincial People's Hospital, Guangdong Academy of Medical Sciences Guangzhou Guangdong China; ^4^ School of Medicine South China University of Technology Guangzhou Guangdong China; ^5^ Southern Medical University Guangzhou Guangdong China; ^6^ Department of Radiology the Affiliated Cancer Hospital of Zhengzhou University, Henan Cancer Hospital Zhengzhou Henan China; ^7^ Department of Radiology The Third Affiliated Hospital of Kunming Medical University, Yunnan Cancer Hospita, Yunnan Cancer Center Kunming Yunnan China

## Abstract

Quantitative features extracted from biopsy digital pathology images can provide predictive information for neoadjuvant chemoradiotherapy (nCRT) in local advanced rectal cancer (LARC) Machine learning technologies are applied to build the digital‐pathology‐based pathology signature The pathology signature is an independent predictor of treatment response to nCRT in LARC

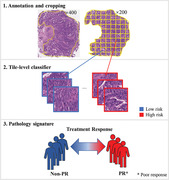

**List of abbreviations**
AUC: area under the curve
CEA: carcinoembryonic antigen
CI: confidence interval
H&E: hematoxylin and eosin
H‐L: Hosmer‐Lemeshow
LARC: locally advanced rectal cancer
LASSO: least absolute shrinkage and selection operator
MRI: magnetic resonance imaging
nCRT: neoadjuvant chemoradiotherapy
PR: poor response
ROC: receiver operating characteristic
ROI: region of interest
SVM: support vector machine
TRG: tumor regression grade
WSI: whole slide images

Colorectal cancer has third‐highest morbidity and second‐highest mortality worldwide. Around one‐third of the tumors are localized in the rectum, and about 70% of rectal cancer is locally advanced rectal cancer (LARC).^1^, ^2^ The standard treatment for LARC involves neoadjuvant chemoradiotherapy (nCRT) followed by surgery.^3^ By downstaging and downsizing the tumor, nCRT increases the chance of subsequent successful R0 resection and sphincter‐preserving surgery, and decrease the chance of local relapse.^4^ However, nCRT may weaken the immune system and cause delayed surgery for patients who cannot benefit from it.^5^, ^6^ Therefore, it is necessary to identify biomarkers for the treatment response to nCRT for LARC, and to pinpoint the patients who will not benefit from it to improve treatment strategy and reduce unnecessary pain and cost.

To predict and monitor the treatment response to nCRT in LARC, several tumor‐related biomarkers have been proposed, including pathological, radiological, clinical, and molecular ones. Certain radiological and molecular markers have shown promise in the response prediction, the reported sensitivity and specificity were limited.^7^ Besides, although some preoperative clinicopathological features like enlarged size and tumor stage have been proposed to predict response to nCRT, but their prediction performance was unstable.^8^, ^9^ Therefore, robust biomarkers with high accuracy still need to be identified and validated.

Biopsy samples are indispensable for the diagnosis of gastrointestinal tumors in current clinical practice. Advances in computerized image processing technology have generated automated histopathological analysis based on the digital whole slide images (WSIs) of biopsy specimens. As a useful approach for tumor diagnosis and prognosis, it has been increasingly investigated in oncology in recent years, with works reported in counting mitoses,^10^ quantifying tumor‐infiltrating immunocyte,^11^ and predicting the grade of tumor differentiation.^12^ Yu et al. selected areas of dense tumor cells in hematoxylin and eosin (H&E) stained WSIs and quantifies features to predict the non‐small cell lung cancer prognosis.^13^ Another study predicted microsatellite instability from the tumor areas of the H&E histology slides in gastrointestinal cancer.^14^ These studies have suggested that WSIs and machine learning approaches can be used to identify and quantify image features beyond simple densities in traditional pathologic interpretation and to explore the potential correlation with the features and treatment response.

For our knowledge, there is no published study on digital‐pathology‐based biomarkers that uses biopsy H&E histology images to predict the treatment response to nCRT in LARC. Therefore, we aim to investigate whether the quantitative features of H&E stained histology slides can predict treatment response.

This study was retrospective and single‐centered. We obtained the approval from the institutional review board of our hospital and observed the Helsinki Declaration and relevant guidelines throughout the work.

A total of 151 LARC patients with adenocarcinomas who received nCRT treatment between January 2013 and June 2018 were recruited by the criteria in Appendix S1. Their baseline clinicopathologic data, including age, gender, pretreatment clinical T and N stage, pretreatment carcinoembryonic antigen (CEA), tumor location, and size, were derived from medical records (Table 1). The tumor location was the distance from the lower edge of colonoscopy to anus, and the tumor size was measured by the length and thickness of tumor from computed tomography imaging. The patients were split randomly into primary and validation datasets according to the ratio of 80%:20%. No significant difference existed in the clinicopathological data between the two datasets (Appendix S2). The complete workflow of data analysis is shown in Figure 1.

**TABLE 1:** Clinical characteristic in the primary and validation datasets

	Primary dataset			Validation dataset		
Characteristic	Non‐PR	PR	*P‐*value	Non‐PR	PR	*P*‐value
Age, mean ± SD	56.0 ± 11.4	55.4 ± 10.9	.465	51.7 ± 11.8	60.4 ±9.18	.012^*^
Gender, No. (%)			.401			.800
Male	38 (62.3%)	42 (71.2%)		8 (72.7%)	14 (70.0%)	
Female	23 (37.7%)	17 (28.8%)		3 (27.3%)	6 (30.0%)	
T staging, No. (%)			.698			.378
T0	0 (0%)	0 (0%)		0 (0%)	0 (0%)	
T1	0 (0%)	0 (0%)		0 (0%)	0 (0%)	
T2	2 (3.3%)	1 (1.7%)		1 (9.1%)	0 (0%)	
T3	23 (37.7%)	26 (44.1%)		4 (36.4%)	7 (35.0%)	
T4	36 (59.0%)	32 (54.2%)		6 (54.5%)	13 (65.0%)	
N staging, No. (%)			.015^*^			.521
N0	6 (9.8%)	17 (28.8%)		2 (18.2%)	4 (20.0%)	
N1	46 (75.4%)	31 (52.5%)		8 (72.7%)	11 (55.0%)	
N2	9 (14.8%)	11 (18.6%)		1 (9.1%)	5 (25.0%)	
CEA level, No. (%)			.134			.724
Normal	30 (49.2%)	38(64.4%)		5 (45.5%)	9 (45.0%)	
Abnormal	31 (50.8%)	21 (35.6%)		6 (54.5%)	11 (55.0%)	
Tumor location (cm)			.313			.298
< 5	36(59.1%)	40 (67.8%)		8 (72.7%)	9 (45.0%)	
5‐10	25 (40.9%)	18 (30.5%)		3 (27.3%)	10 (50.0%)	
≥10	0 (0%)	1 (1.7%)		0 (0.0%)	1 (5.0%)	
Length of tumor (cm), mean ± SD	4.87 ± 1.88	4.92 ± 1.52	.483	4.56 ± 1.19	4.89 ± 1.70	.917
Thickness of tumor (cm), mean ± SD	1.77 ± 0.69	1.76 ± 0.65	.945	1.27 ± 0.38	1.76 ± 0.53	.03^*^
Pathology score, median (interquartile range)	0.128 (0.089 to 0.321)	0.891 (0.626 to 0.929)	<.001^*^	0. 207 (0.130 to 0.483)	0.814(0.481 to 0.874)	<.001^*^

Note. *P*‐value is derived from the univariable association analyses between each of the clinicopathological variables and treatment response. The clinical characters were the data from the initial diagnosis. The threshold value for CEA level was ≤5 ng/mL and >5 ng/mL according to the universally normal range used.

Abbreviations: CEA, pre‐treatment carcinoembryonic antigen; SD, standard deviation.

* *P *< .05.

**FIGURE 1:**
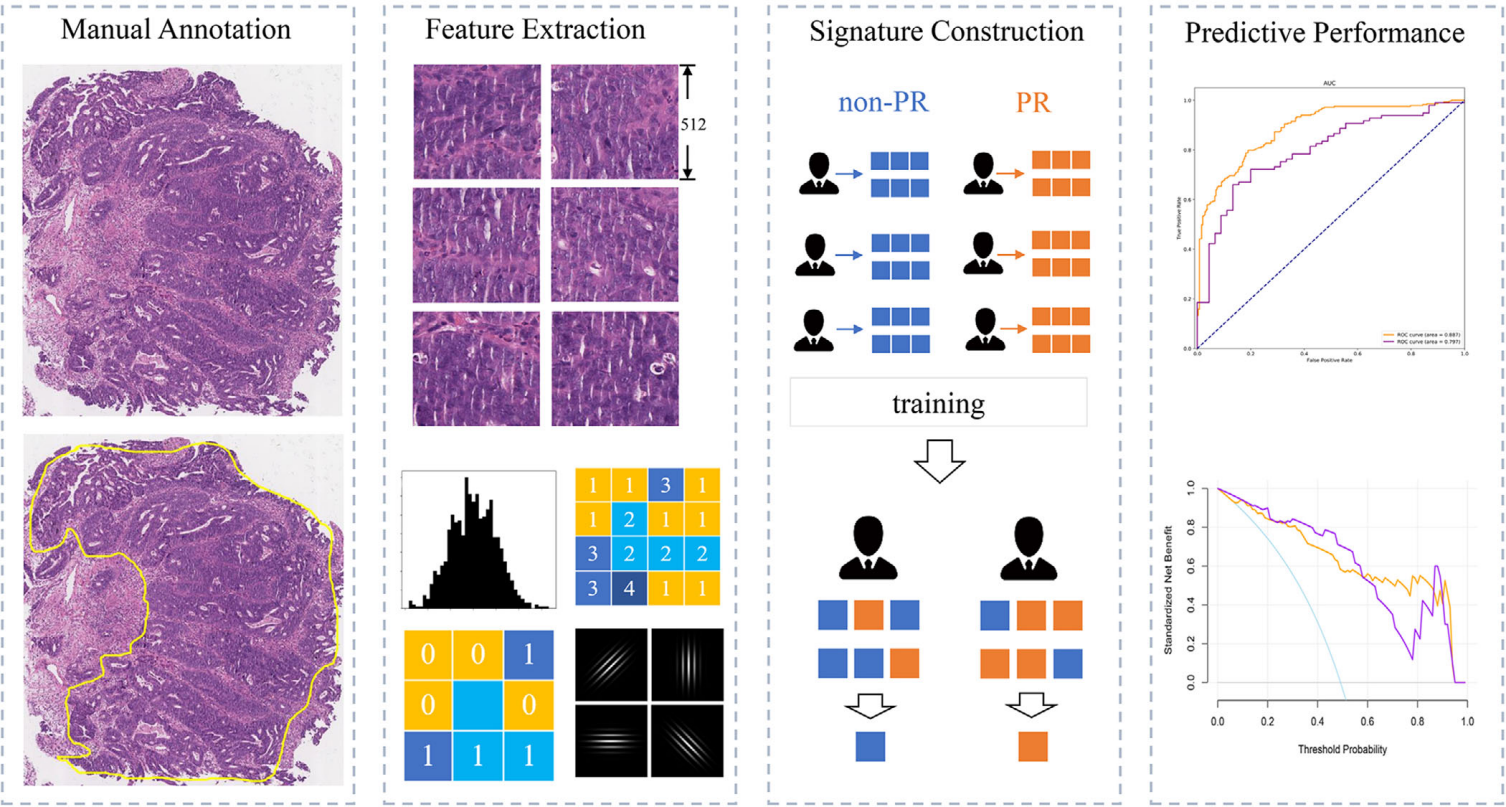
Pathology signature construction in hematoxylin and eosin stained whole slide images (WSIs). With manually annotated tumor areas, we cropped WSIs into tile images; quantitative features were extracted and reduced from the selected patches of tumor cell dense area. Next, we built a tile‐level classifier via a support vector machine (SVM) model, and then the pathology signature was constructed with a logistic regression model. Finally, the predictive power of the signature was evaluated

The nCRT regimen is provided in Appendix S3. The treatment response to nCRT was evaluated according to tumor regression grade (TRG).^6^ TRG consists of 0, 1, 2, and 3, whose details are described in Appendix S4. We divided the treatment response into two categories, with TRG 2/3 being poor response (PR), where patients did not benefit from nCRT, and TRG 0/1 being non‐poor response (non‐PR). There were no significant differences between non‐PR and PR groups in all included clinical characteristics concerning age, gender, pretreatment clinical T stage, and CEA‐level except clinical N stage (*P* = .015 and *P* = .521 in primary and validation datasets, respectively) and age (*P* = .023 in validation dataset) (Table 1).

All the H&E WSIs used in this study were obtained from Yunnan Cancer hospital. The sample collection process is described in Appendix S5. The regions of interest (ROIs) encompassing tumor regions at ×400 magnification were roughly annotated by an experienced pathologist (with 10 years of clinical experience) by using Aperio ImageScope and were confirmed by another pathologist (with 25 years of clinical experience). To make computation feasible, the ROIs were cropped into minimum bounding rectangle images. Those images were downsampled to lower resolution of ×200 magnification and tiled into 512 × 512 pixels without overlapping. From those tiles, the tiles in the regions of the tumor cells were selected by the first pathologist and were confirmed by second. In the end, a total of 667 tiles were selected (525 from the primary cohort and 142 from the validation one).

A total of 104 texture features were extracted from each of the selected tiles,^15^, ^16^ and the most helpful predictive features were selected by using the least absolute shrinkage and selection operator (LASSO) method with 10‐fold cross‐validation from the primary cohort (Figure 2A,B). LASSO has sparse solutions to reduce the dimensionality of the data.^17^ At last, 17 potential predictors were distilled from the 104 texture features in the primary cohort (Appendix S6).

**FIGURE 2:**
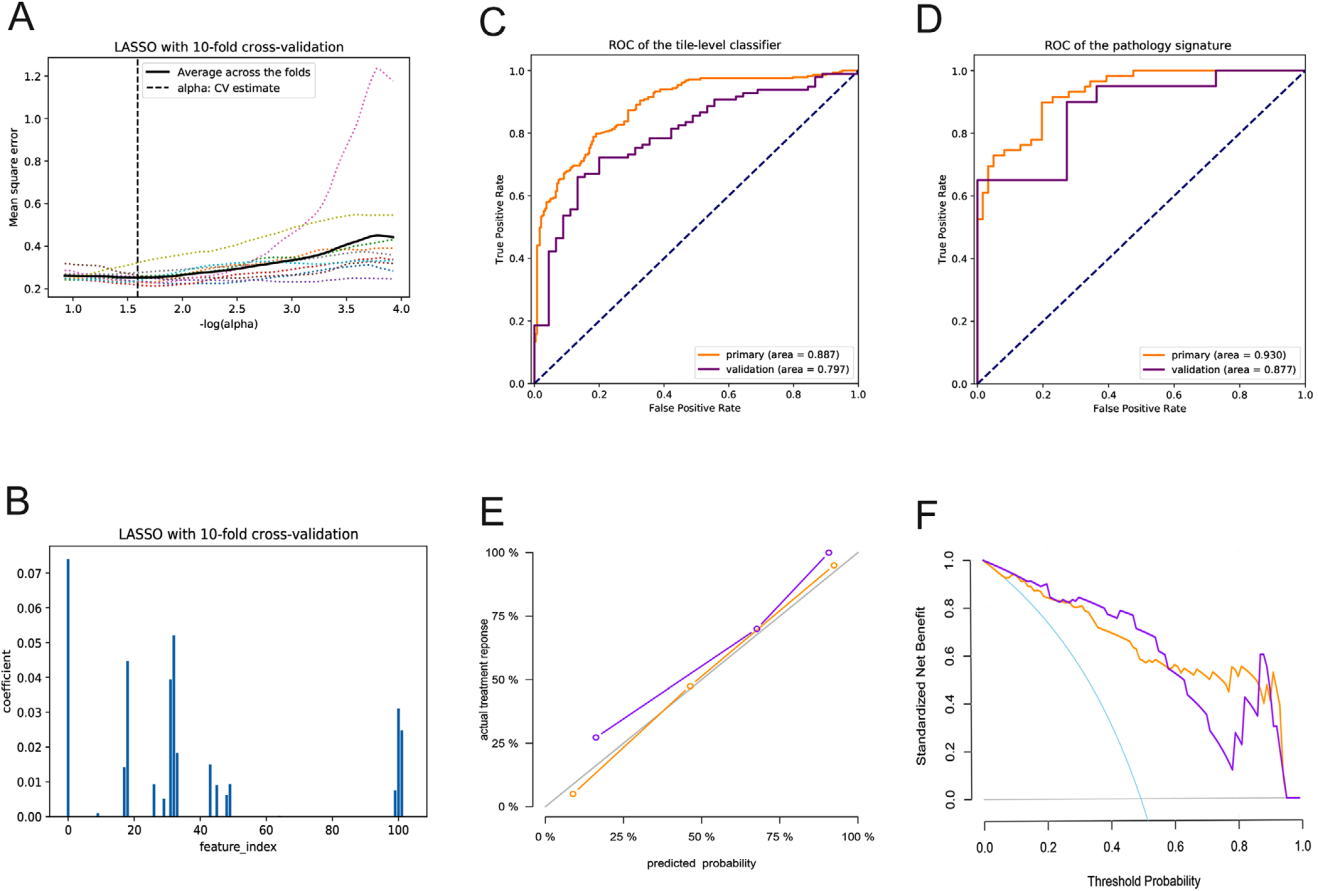
Texture feature selection and pathology signature's performance. A, The parameter alpha selection in the least absolute shrinkage and selection operator (LASSO) model used the 10‐fold cross‐validation. B, LASSO coefficient profiles of the 104 texture features using 10‐fold cross‐validation. C, the receiver operating characteristics (ROC) curve of the tile‐level classifier in the primary and validation dataset. D, ROC curve of the pathology signature in the primary and validation datasets. E, calibration curves of the pathology signature in the primary and validation datasets. F, Decision curve analysis for the pathology signature in the primary and validation datasets

The pathology signature was built as follow steps. The first step was to build a support vector machine (SVM) model with a radial basis function kernel, namely tile‐level classifier, based on selected features to determine whether the selected tiles were classified. The second step was to compute the mean of all selected tiles’ probabilities that the tile‐level classifier output in each patient, and then to regard the mean as each patient's characteristic for further statistical analyses. The third step was to calculate a pathology score via the logistic regression method to discriminate pathology‐reported PR status based on the characteristic for each patient in this study (Appendix S7 and S8). The multivariable logistic regression analysis, including age, gender, pretreatment clinical T and N stage, CEA level as well as the pathology score, showed that the pathology score was the only independent predictor (Appendix S7).

To evaluate the discriminative power of the pathology signature, the receiver operating characteristics (ROC) curve was applied. The tile‐level classifier for distinguishing PR from non‐PR produced an area under the ROC curve (AUC) of 0.887 (95% confidence interval (CI), 0.858‐0.916), and 0.797 (95% CI, 0.718‐0.866) in the primary and validation datasets, respectively (Figure 2C). The pathology signature provided an AUC of 0.930 (95% CI, 0.883‐0.966), and 0.877 (95% CI, 0.719‐0.97) in the two datasets, respectively (Figure 2D). Meanwhile, we use accuracy, sensitivity, specificity, and F1 score to assess the signature performance (Table 2). The calibration of the signature was evaluated by using the calibration curves and Hosmer‐Lemeshow (H‐L) test (Figure 2E). The clinical usefulness of the signature was determined by using the decision curve analysis, which could quantify the net benefits at different threshold probabilities in the primary and validation datasets (Figure 2F). The H‐L test generated a non‐significant statistic (*P* = .332 in primary dataset, *P* = .213 in validation dataset), suggesting no departure from a good fit.

**TABLE 2:** Performance of pathology signature

	Accuracy(95%CI)	Sensitivity(95% CI)	Specificity(95% CI)	F1‐score(95% CI)	AUC(95%CI)
TL‐p	0.790 (0.756‐0.82)	0.760 (0.714‐0.811)	0.826 (0.778‐0.872)	0.796 (0.760‐0.833)	0.887 (0.858‐0.916)
TL‐v	0.732 (0.655‐0.803)	0.753 (0.663‐0.833)	0.688 (0.553‐0.821)	0.793 (0.720‐0.853)	0.797 (0.718‐0.866)
P‐p	0.792 (0.708‐0.858)	0.780 (0.661‐0.873)	0.803 (0.692‐0.897)	0.786 (0.685‐0.855)	0.930 (0.883‐0.966)
P‐v	0.710 (0.548‐0.871)	0.700 (0.476‐0.889)	0.727 (0.444‐1.0)	0.757 (0.564‐0.878)	0.877 (0.719‐0.97)

Abbreviations: CI, confidence interval; P‐p, the performance of primary dataset in pathology signature; P‐v, the performance of validation dataset in pathology signature; TL‐p, the performance of primary dataset in tile‐level classifier; TL‐v, the performance of validation dataset in tile‐level classifier

The tools and methods of the statistical analysis are shown in Appendix S9. The reported statistical significance levels were all two‐sided, with statistical significance set at 0.05.

In this study, we presented a digital‐pathology‐based signature to predict treatment response to nCRT in LARC. We showed that quantitative features extracted from H&E histology slides could provide predictive information for treatment response.

The digital‐pathology‐based signature we proposed has advantages over the previous methods or complements them. Magnetic resonance imaging (MRI) is the major method to predict the treatment response to nCRT in LARC.^18^, ^19^ Zhou et al. predicted non‐response to neoadjuvant therapy in LARC by using pretreatment MRI images and achieved an AUC of 0.773.^19^ We yielded an AUC of 0.877 by using pathological images, demonstrating a better prediction performance than that achieved by Zhou et al. Besides, Nie et al. used pretreatment multiparametric MRI images for nCRT efficacy evaluation and obtained an AUC of 0.89,^5^ but the approach is limited partly because some patients cannot receive MRI examinations. In such cases, our method can serve as an alternative.

Our work provides a new and easy‐to‐apply method based on the biopsy‐acquired H&E slides for the treatment response prediction in LARC, which could be well suited for routine clinical practice. The previous studies showed the quantitative image features could be mined through approaches of digital pathology and machine learning, but their clinical applications faced obstacles, like the heavy annotation workload.^20^ However, the pathology signature in our study required a small amount of annotating work, namely circling parts of tumor areas and selecting a few tiles. Besides, the tiles in our study were cropped from the ×200 magnification WSIs that is different from the previous study using the ×400 magnification WSIs.^13^ The tiles from the ×400 magnification WSIs may provide more details but have a narrow field of view, while the tiles from the ×200 magnification WSIs could reflect a wider range of tumor tissue patterns and make more pathological information comprehensible.^21^

A key part of our image processing approach is to select tiles of dense tumor cell areas. Because the normal rectum area is composed predominantly of regular epithelium and normal cell with typical morphological characteristics, the areas of dense tumor cell show exhibit pathological changes, such as different cell nucleus and gland morphology, and image feature extraction from the tiles of the tumor area is potential to be biologically informative.^22^ The strategy of using representative patch samples was adopted when selecting tiles to avoid the time‐consuming way of utilizing all image patches of each WSIs.^23^

Some limitations exist in our study. First, the size of patient samples was relatively small considering the large number of predictors. Second, the quality of H&E biopsy slides may not be consistent in some cases, where the slide quality may have been affected by where the material was taken from and which microscopical section was chosen. Hence measures are needed to control slide quality, and a large, independent, and prospective multicenter validation queue is needed to examine whether the proposed model can be extended and the clinical potential be transformed.

In conclusion, we developed a pathology signature as a new method to predict the treatment response to nCRT in LARC, which can help make personalized treatment plans and improve outcomes for patients.

## ETHICS APPROVAL AND CONSENT TO PARTICIPATE

This study follows the Declaration of Helsinki and good clinical practice guidelines, and was approved by Medical Ethics Committee of Guangdong Provincial People's Hospital.

## CONFLICT OF INTEREST

The authors declare that they have no potential conflicts of interest.

## Supporting information

Supporting Information
