# Supplementary material for: Predicting treatment response to neoadjuvant chemoradiotherapy in local advanced rectal cancer by biopsy digital pathology image features
Source: Clin Transl Med. 2020 Jun 28;10(2):e110. doi: 10.1002/ctm2.110 (PMC7403709; doi:10.1002/ctm2.110)
Supplement: Supplementary file 1 — Supporting Information [file CTM2-10-e110-s001.docx]

**Appendices**

**Appendix A1: Patient Recruitment**

The inclusion criteria were as follows: (1) locally advanced disease determined by pretreatment TNM stage (T3/T4, and/or positive nodal status); (2) received pathological biopsy and had Hematoxylin and eosin (H&E) stained slide; (3) received no treatment before neoadjuvant chemotherapy and had completed neoadjuvant chemotherapy; (4) tumor regression grade (TRG) was confirmed by pathologic examination after surgical treatment. The exclusion criteria were as follows: (1) no or unclear TRG after surgical treatment; (2) no H&E stained slide; (3) no tumor area in H&E stained slides.

**Figure S1 Pathway for patient recruitment in the study**

Archive data of patient with locally advanced rectal cancer from October 2012 to March 2018

(n = 251)

Excluded for following reasons (n=44):

lacking biopsy slides information (n=8)

and lack of H&E-stained slide (n=36)

Biopsy H&E stained slides eligibility(n=207)

Patient included in the retrospective (n=151) split randomly into two cohorts

uinto

There was no TRG (n = 31), or TRG was unclear (n = 19) after surgical treatment

No tumor area in H&E stained slides (n = 6)

Primary cohort

(n = 120)

Validation cohort

(n = 31)

**Appendix A2: Demographic comparison between primary and validation cohorts**

**Table S1 Demographic comparison between primary and validation cohort**

| **Characteristic** | **Primary** | **Validation** | **p-value** |
| --- | --- | --- | --- |
| Age, mean (SD) | 55.8(11.2) | 57.1 (10.7) | 0.732 |
| Gender, No (%) |  |  | 0.810 |
| Male | 80 (66.7%) | 22 (71.0%) |  |
| Female | 40 (33.3%) | 9 (29.0%) |  |
| Clinical T staging, No (%) |  |  | 0.471 |
| T0 | 0(0%) | 0(0%) |  |
| T1 | 0(0%) | 0(0%) |  |
| T2 | 3 (2.5%) | 1 (3.2%) |  |
| T3 | 49 (40.8%) | 11 (35.5%) |  |
| T4 | 68 (56.7%) | 19 (61.3%) |  |
| Clinical N staging, No (%) |  |  | 0.929 |
| N0 | 23 (19.2%) | 6 (19.4%) |  |
| N1 | 77 (64.2%) | 19 (61.3%) |  |
| N2 | 20 (16.7%) | 6 (19.4%) |  |
| CEA level, No (%) |  |  | 0.345 |
| Normal | 68 (56.7%) | 14 (45.2%) |  |
| Abnormal | 52 (43.3%) | 17 (54.8%) |  |
| Treatment response, No (%) |  |  | 0.186 |
| Non-PR | 61 (50.8%) | 11 (35.5%) |  |
| PR | 59 (49.2%) | 20 (64.5%) |  |
| Tumor location |  |  | 0.449 |
| < 5 | 76 | 17 |  |
| 5 - 10 | 46 | 13 |  |
| ≥10 | 1 | 1 |  |
| Length of tumor (cm),  mean (SD) | 4.89 (1.71) | 4.77 (1.54) | 0.895 |
| Thickness of tumor (cm),  mean (SD) | 1.76 (0.670) | 1.59 (0.539) | 0.251 |

Note. Baseline data comparison is shown in the above table. The length of tumor was the length of the initially diagnosed lesion in computed tomography (CT) imaging. The thickness of tumor was the thickness of the initially diagnosed CT lesion.

Abbreviation: CEA, pretreatment carcinoembryonic antigen. PR, poor response.

**P* < 0.05.

**Appendix A3: Neoadjuvant chemoradiotherapy treatment (nCRT)**

The nCRT regimen before November 2016 was different from that after the November 2016. A total of 73 patients between January 2013 and October 2016 received neoadjuvant concurrent chemoradiotherapy. A total of 78 patients between November 2016 and June 2018 received two cycles of chemotherapy followed concurrent chemoradiotherapy before surgery. The neoadjuvant concurrent chemoradiotherapy regimen: Capecitabine/long-course radiotherapy (45 – 50 Gy in 25 – 28 fractions) or bolus 5-FU/leucovorin/long-course radiotherapy (45–50 Gy in 25 – 28 fractions). The interval between concurrent chemoradiotherapy and surgery was 6 - 8 weeks. The chemotherapy regimen before the surgery was the same as that of the concurrent chemoradiotherapy. The interval between chemotherapy and surgery was 2 - 3 weeks.

**Appendix A4: Tumor regression grade (TRG)**

According to literature^1^, the grades of the TRG are followed:

TRG 0, complete response. no viable cancer cells;

TRG 1, near-complete response, single cells or rare small groups of cancer cells;

TRG 2, partial response, residual cancer with evident tumor regression, but more than single cells or rare small groups of cancer cell;

TRG 3, poor or no response, extensive residual cancer with no evident tumor regression.

**Appendix A5:** **Collection and processing of patient histological samples**

The slides were captured at ×400 magnification and digitized on Leica Aperio AT2 scanners, in which a single pixel represented 0.252µm × 0.252µm of the actual size. These images were in .svs file format.

A total of 5 tiles of tumor cell dense area were selected from each patient by an experienced pathologist and confirmed by another. But some patients had just one or two tiles because the number of candidate tiles was limited in these cases, where biopsy specimens contained only small tumor areas with even smaller annotation. Figure 2S shows the inclusion criteria of selected tiles.


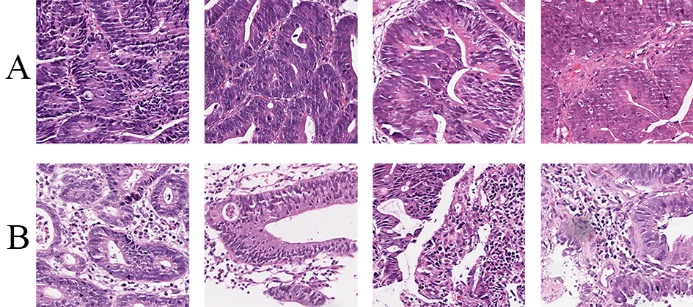


**Figure 2S** Inclusion criteria of the selected patch images (Hematoxylin and Eosin, magnification ×200). A, patches of dense tumor cell areas. B, excluded tiles with large background areas, tissue of other types, and artificial damage or pollution

**Appendix A6 Image feature extraction and feature selection**

Texture features used in this study are listed as follows.

**TABLE S2 Texture features of pathology tiles**

| **Texture type** | **Texture name** |
| --- | --- |
| Perception-like features | coarseness  contrast  directionality  line-likeness  roughness |
| Percentile | prctile_25  prctile_250  prctile_500  prctile_750  prctile_975 |
| Histogram-higher | Moment6~moment10 |
| Histogram-lower | Mean  Variance  Skewness  Moment5  Kurtosis |
| Lbp | Lbp1~lbp38 |
| Gabor | Gabor1~Gabor6 |
| GLCM | Energy  Contrast  Correlation  Homogeneity  Variance  Sum Average  Entropy |
| GLRLM | Short Run Emphasis (SRE)  Long Run Emphasis (LRE)  Gray-Level Non-uniformity (GLN)  Run-Length Non-uniformity (RLN)  Run Percentage (RP)  Low Gray-Level Run Emphasis (LGRE)  High Gray-Level Run Emphasis (HGRE)  Short Run Low Gray-Level Emphasis (SRLGE)  Short Run High Gray-Level Emphasis (SRHGE) Long Run Low Gray-Level Emphasis (LRLGE)  Long Run High Gray-Level Emphasis (LRHGE) Gray-Level Variance (GLV)  Run-Length Variance (RLV) |
| GLSZM | Small Zone Emphasis (SZE)  Large Zone Emphasis (LZE)  Gray-Level Non-uniformity (GLN)  Zone-Size Non-uniformity (ZSN)  Zone Percentage (ZP)  Low Gray-Level Zone Emphasis (LGZE)  High Gray-Level Zone Emphasis (HGZE)  Small Zone Low Gray-Level Emphasis (SZLGE)  Small Zone High Gray-Level Emphasis (SZHGE)  Large Zone Low Gray-Level Emphasis (LZLGE)  Large Zone High Gray-Level Emphasis (LZHGE)  Gray-Level Variance (GLV)  Zone-Size Variance (ZSV) |
| NGTDM | Coarseness  Contrast  Busyness  Complexity  Strength |

Finally, the least absolute shrinkage and selection operator (LASSO) method was applied to reduce the feature dimension.

The LASSO formula,

$$\sum_{i}^{N} (1/(2*N))*\left[ y_{i}-\left( \sum w*x \right) \right]^{2}+\mathrm{alpha}* \sum|w|$$

Where $y_{i}$ is the observed value of patients, $N$ is the number of samples, $w$ is the model parameters, $x$ is the features of patients, and $alpha$ is the regularization parameter.

The selected features were prctile_25, Moment10, perceptual_Directionality, perceptual_LineLikeliness, LBP7, LBP10, LBP12, LBP13, LBP14, LBP24, LBP26, LBP29, LBP30, GLCM_Energy, NGTDM_Coarseness, NGTDM_Contrast and NGTDM_Busyness.

**Appendix A7: Pathology signature building**

Pathology signature construction included three parts.

The tile-level classifier was built via a support vector machine (SVM) model with a radial basis function kernel. The SVM model with a radial basis function kernel transformed the original input space to a new space. $a_{i}, \gamma,b$ were the SVM parameter.

The radial basis function kernel,

$${k(x,x^{'})= e}^{-\gamma{\parallel x-x^{'}\parallel}^{2}}$$

The SVM model,

$$f\left( x \right)=sign\left( \sum_{i=1}^{N} a_{i}y_{i}k(x_{i},x)+b \right)$$

Next, we computed the mean of all selected tiles’ probabilities that the tile-level classifier output in each patient, and then regarded the mean as each patient’s characteristic for further statistical analyses. M is the number of tiles for each patient.

$${mean}_{f\left( x \right)}=\frac{1}{M}*\sum_{i=1}^{M} f(x)$$

The logistic regression model was used to construct the pathology signature based on the average value. $\theta$ is the weight of our eventual model in the following formulas.

$$h_{\theta}\left( x \right)=\sum_{i=1}^{n} \theta_{i}x, n=1$$

$$g\left( x \right)= \frac{1}{1+e^{h_{\theta}(x)}}$$

Eventually, a pathology score was computed via the pathology signature.

A number of candidate predictors were used to start multivariable logistic regression analysis. They included age, gender, pretreatment clinical T and N stage, CEA level as well as the pathology score. The analysis results showed the pathology score was the only independent predictor (Table S3).

**Table S3 Risk Factors for treatment response in local advanced rectal cancer**

| Characteristic | OR (95%CI) | *P-*value |
| --- | --- | --- |
| Age | 1.018 (0.971, 1.070) | 0.462 |
| Clinical N stage | 1.013 (0.374, 2.745) | 0.980 |
| Thickness of tumor | 0.902 (0.380, 2.252) | 0.819 |
| Pathology score | 3.336 (2.361, 5.168) | < 0.001* |

Note. Abbreviation: CI, confidence interval. OR, odd ratio.

**P* < 0.05

**Appendix A8: Calculation formula of pathology signature**

The calculation formula of the tile-level classifier,

$${k(x,x^{'})= e}^{-0.009{\parallel x-x^{'}\parallel}^{2}}$$

$$f\left( x \right)=sign\left( \sum_{i=1}^{N} y_{i}k(x_{i},x)+0.216 \right)$$

The calculation formula of the logistic regression model,

$$g\left( x \right)= \frac{1}{1+e^{2.463*x}}$$

**Appendix A9: Statistical analysis**

Independent Samples t-test, Chi-Squared test, or Mann-Whitney U test was chosen when appropriate to check the difference for continuous variables. Chi-Squared test was applied to make a comparison for categorical ones. Feature-extracting algorithms and image processing were performed by using Matlab R2019a software. Statistical analysis was done with Python (version 3.7) and R software (version 3.6, http://www.Rproject.org). Python packages, including “numpy”, “pandas”, “spicy”, “scikit-learn”, and “matplotlib”, were mainly used for the pathology signature and significance analysis. The calibration curve and the decision curve were plotted by “riskregression” package and “DecisionCurve” package in R language respectively.

Reference:

1. Song C, Chung JH, Kang SB, et al. Impact of Tumor Regression Grade as a Major Prognostic Factor in Locally Advanced Rectal Cancer after Neoadjuvant Chemoradiotherapy: A Proposal for a Modified Staging System. *Cancers (Basel).* 2018;10(9):319.
